# Emerging evidence from China indicates SARS- COV-2 may be associated with newly diagnosed Hashimoto’s thyroiditis: a clinical study of 54 patients with COVID-19 complicated by newly diagnosed disease

**DOI:** 10.3389/fendo.2026.1749196

**Published:** 2026-03-09

**Authors:** Jingyu Hou, Yu Zhang, Boyu Zhang, Sen Zhao, Han Wu, Xingxing Li, Min Li, Yongzhong Guo, Ning Song

**Affiliations:** 1 Department of Infectious Diseases of The Second Hospital of Hebei Medical University, Shijiazhuang, Hebei, China; 2 Department of Respiratory and Critical Care Medicine, Xuzhou Central Hospital, Xuzhou, Jiangsu, China

**Keywords:** autoimmune thyroid disease, COVID-19, Hashimoto’s thyroiditis, thyroid function, viral clearance time

## Abstract

**Background:**

To date, 11 cases of COVID-19-associated newly diagnosed Hashimoto’s thyroiditis (HT) have been reported. To investigate the clinical characteristics and influencing factors of Corona Virus Disease 2019 (COVID-19) complicated by newly diagnosed Hashimoto’s thyroiditis (HT) and to inform clinical management.

**Method:**

A total of 369 COVID-19 patients were enrolled and divided into three groups: Negative for Antibodies (NA group, n=288), HT group (n=54), and Thyroid Autoimmune (TA group, n=27). The clinical characteristics and influencing factors of the HT group were analyzed. Data analysis was performed using SPSS 27.0 statistical software.

**Results:**

Patients in the HT group were older than those in the NA group [72.5 (63.5, 79.0) vs. 69 (58.3, 76.0) years, P = 0.031]. Fatigue was significantly more common in the HT group compared to both the TA group and the NA group (53.7% vs. 22.2%, and 53.7% vs. 24.0%, all P<0.001). The incidence rates of subclinical hyperthyroidism and hypothyroidism in the HT group were significantly higher than in the NA group (29.6% vs. 13.9%, P = 0.020; 7.4% vs. 1.7%, P = 0.017). Multivariate analysis identified prolonged viral clearance time (OR = 1.136) as an independent risk factor for COVID-19 complicated by HT. Advanced age (OR = 0.944) and low FT3 (OR = 4.233) were identified as independent risk factors for increased mortality risk in COVID-19 patients.

**Conclusion:**

COVID-19 patients complicated by HT are typically older, with fatigue being a distinguishing clinical manifestation. They are more prone to thyroid dysfunction, with subclinical hyperthyroidism being the predominant early thyroid function abnormality. Prolonged viral clearance time was identified as a factor associated with newly diagnosed HT.

## Introduction

The novel coronavirus infection (COVID-19), caused by the Severe Acute Respiratory Syndrome Coronavirus-2 (SARS-CoV-2), has significantly impacted global public health (1). COVID-19 targets not only the upper respiratory tract and lungs but also multiple organ systems, including the cardiovascular, gastrointestinal, nervous, and endocrine systems (2). Accumulating evidence suggests an association between COVID-19 and the onset of multiple autoimmune diseases, including autoimmune thyroid disease (AITD) (3). HT, the most prevalent AITD (4), represents the primary cause of impaired thyroid function (5). HT is pathologically defined by lymphocyte infiltration of the thyroid gland and elevated levels of thyroid-specific antibodies, such as Thyroid Peroxidase Antibody (TPOAb)and Thyroglobulin Antibody (TgAb) (6).

The pathogenesis of HT results from the interaction of genetic susceptibility and environmental factors, with viral infections representing a key environmental trigger (7). For instance, enterovirus, parvovirus, herpesvirus, and hepatitis C virus have been epidemiologically linked to autoimmune thyroiditis (8). The SARS-CoV-2 receptor, angiotensin-converting enzyme 2 (ACE2), and its cofactor transmembrane protease serine 2 (TMPRSS2), are expressed on thyroid follicular epithelial cells, indicating a structural basis for thyroid invasion by SARS-CoV-2 (9). The spike (S) protein of SARS-CoV-2 shares structural and antigenic homology with thyroid peroxidase (TPO), potentially inducing immune cross-reactivity and promoting TPO-directed autoimmunity (10). Studies demonstrate that thyroid dysfunction may arise during the acute phase of COVID-19 (11), with elevated levels and positivity rates of thyroid autoantibodies (e.g., TPOAb) observed in COVID-19 patients compared to the general population (12, 13). To date, 11 cases of newly diagnosed HT following COVID-19 infection have been documented globally (14–23). Evidence suggests an association between AITD and COVID-19 (24), while HT is further linked to a significantly increased risk of post-COVID-19 thyroid cancer and pulmonary fibrosis (25, 26). Thus, elucidating the relationship between COVID-19 and HT holds substantial clinical and research implications.

## Participants and methods

### Study subjects

**Figure 1:**
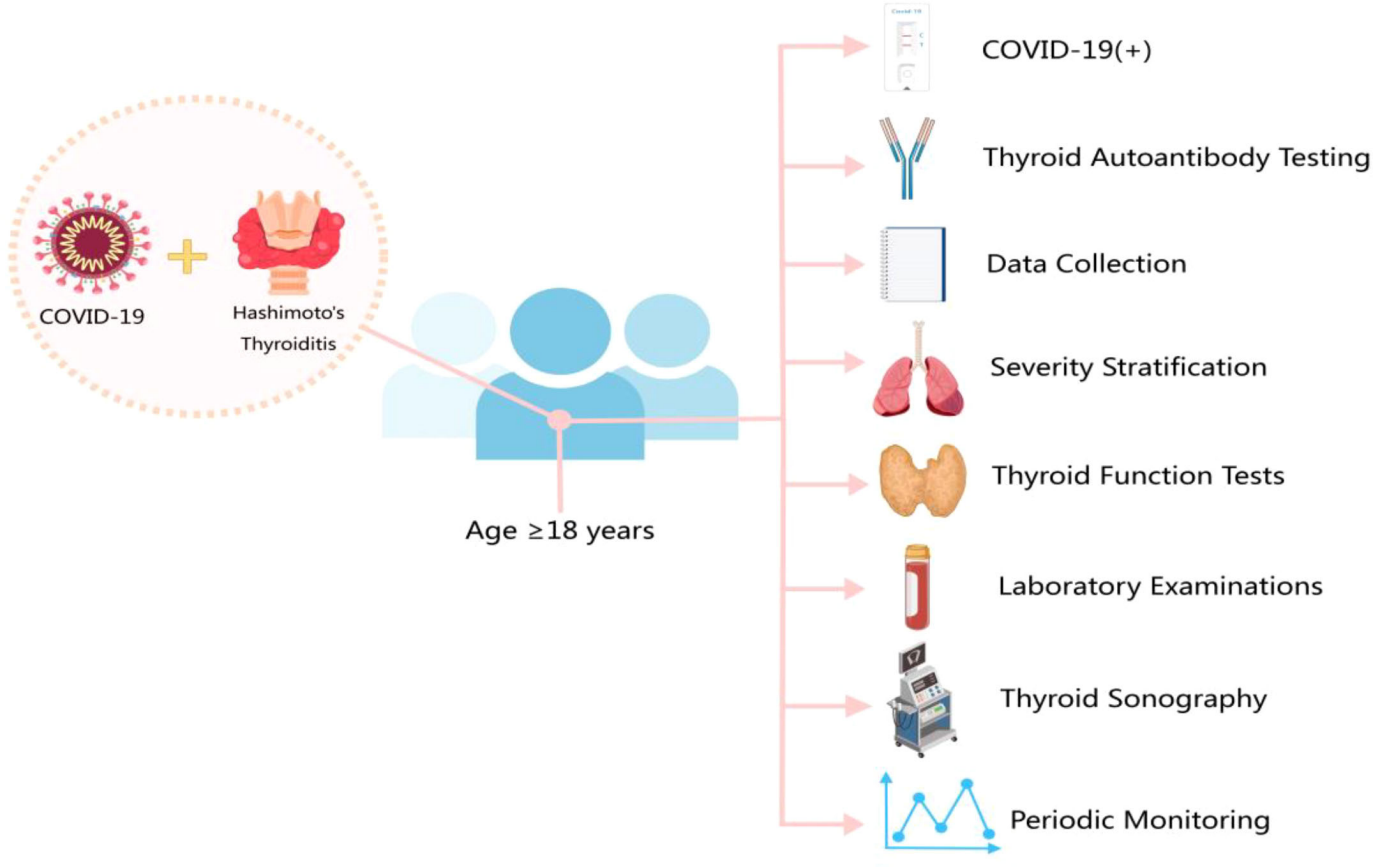
Research flowchart.

A retrospective cohort study was conducted using the electronic medical record system of the Second Affiliated Hospital of Hebei Medical University from December 25, 2022, to June 24, 2024, to identify cases diagnosed with COVID-19. A detailed research workflow for adult COVID-19 patients with Hashimoto’s thyroiditis is illustrated in **Figure 1**. Eligible subjects were selected based on predefined inclusion and exclusion criteria.

#### Inclusion criteria

1. Participants aged ≥18 years old;
2. Thyroid autoantibodies (TPOAb and TgAb) tested upon admission;
3. Complete clinical data available.

#### Exclusion criteria

1. Age <18 years;
2. History of thyroid disease or pre-admission use (within 1 month prior to hospitalization) of:
  • Thyroid medications (including hormone replacement therapy)
  • Heparin
  • Glucocorticoids
  • Interferons
  • Hydroxychloroquine (27);
3. Autoimmune diseases;
4. Pregnancy;
5. Incomplete clinical data.

Note: Drugs were excluded if used within 1 month prior to hospitalization because long-term use of glucocorticoids, interferons, or hydroxychloroquine may modulate immune function and thyroid hormone metabolism, potentially confounding the association between SARS-CoV-2 infection and *de novo* thyroid autoimmunity. Patients who initiated these drugs >24 hours after admission were not excluded, as their use was part of in-hospital COVID-19 treatment and recorded as a confounding variable in regression analyses.

All enrolled patients had received at least two doses of COVID-19 vaccine (predominantly inactivated vaccines, administered according to standard clinical vaccination protocols), with the final dose administered at least one month prior to COVID-19 infection, and none had received any vaccine within two weeks before infection.

#### Study arm assignment

Chinese HT diagnostic criteria (2008 edition): 1. Diffuse goiter with firm consistency, particularly involving isthmus or pyramidal lobe enlargement, irrespective of thyroid function status. 2. Seropositivity for TPOAb and TgAb, which confirms the diagnosis. 3.During pre-hypothyroid stages, the presence of both TPOAb and TgAb serves as the sole diagnostic criterion for HT (6). Elevated TPOAb or TgAb levels represent significant indicators of thyroid autoimmunity (TA) (28). Cases were grouped as:

HT group (n=54): Requiring strict adherence to the Chinese HT diagnostic criteria (2008 edition) (diffuse goiter with dual positivity for TPOAb/TgAb, or dual antibody positivity during the pre-hypothyroid stage).

TA group (n=27): Elevated levels of TPOAb and/or TgAb (regardless of whether they meet the diagnostic criteria for HT).

Negative antibodies (NA) group (n=288): Both TPOAb and TgAb are within the normal range, with no evidence of thyroid autoimmunity.

#### Clinical classification of COVID-19

COVID-19 severity was classified per China’s Diagnosis and Treatment Guidelines for Novel Coronavirus Infection (10th edition) (29):

•Mild: Predominantly upper respiratory tract infection symptoms including dry throat, sore throat, cough, and fever.

•Moderate: Persistent high fever (>3 days) and/or cough/dyspnea, with respiratory rate <30 breaths/min and resting SpO_2_ >93% on room air, plus characteristic CT findings of COVID-19 pneumonia.

•Severe: Any of: 1) Tachypnea (≥30 breaths/min); 2) Resting SpO_2_ ≤93% on room air; 3) PaO_2_/FiO_2_ ≤300 mmHg; or 4) Radiographic progression (>50% lesion expansion within 24–48 hours).

•Critical: Meeting any of: 1) Respiratory failure requiring mechanical ventilation; 2) Shock; or 3) Multi-organ failure necessitating ICU care.

For analysis, cases were grouped as: Non-severe (mild/moderate, n=260) and Severe (severe/critical, n=109).

#### Thyroid function status

Thyroid function status was categorized into six groups (11):

1. Euthyroidism;
2. Non-thyroidal illness syndrome (NTIS) - defined as normal Thyroid Stimulating Hormone (TSH)with either: elevated/normal Free Thyroxine (FT4) and low Free Triiodothyronine (FT3), or low FT4 and FT3;
3. Subclinical hypothyroidism;
4. Subclinical hyperthyroidism - low TSH with normal FT4 and FT3;
5. Overt hypothyroidism - elevated TSH with low FT4 and/or FT3;
6. Overt hyperthyroidism - low TSH with elevated FT3 and/or FT4.

#### Thyroid autoantibody and function testing strategy

All hospitalized COVID-19 patients admitted to the Second Hospital of Hebei Medical University between December 25, 2022, and June 24, 2024, were routinely screened for thyroid function (TSH, FT3, FT4) and thyroid autoantibodies (TPOAb, TgAb) upon admission, except for those with explicit contraindications (e.g., acute hemodynamic instability requiring emergency intervention, known severe coagulation disorders precluding blood sampling). The screening was part of the hospital’s standardized COVID-19 management protocol for hospitalized patients, aiming to assess multi-organ involvement (including endocrine system) during acute infection.

Thyroid autoantibodies (TPOAb, TgAb) were measured usinga[analytical platform name is Roche Cobas e601 electrochemiluminescence immunoassay analyzer]. The measurable ranges were: TPOAb, 0.6~1300 IU/mL; TgAb, 0.1~1000 IU/mL. The positive cut-off values were defined according to the reagent specifications: >60 IU/mL for TPOAb and >60 IU/mL for TgAb.

### Data collection

#### Baseline data and hospitalization records

The following data were collected upon admission: gender, age, body mass index (BMI), clinical symptoms and signs, underlying comorbidities (hypertension, diabetes mellitus, cardiovascular disease, chronic obstructive pulmonary disease [COPD], malignancy), personal history (including smoking status), length of hospital stay, ICU admission rate, and clinical outcomes.

#### Laboratory test results

The following laboratory parameters were assessed: complete blood count (CBC) parameters: white blood cell count (WBC), neutrophil count (NEUT), lymphocyte count (LYMPH), and platelet count (PLT); inflammatory markers: neutrophil-to-lymphocyte ratio (NLR), platelet-to-lymphocyte ratio (PLR), high-sensitivity C-reactive protein (hs-CRP), D-dimer (D-D), interleukins (IL-6, IL-10, IL-17), and tumor necrosis factor-alpha (TNF-a); time to SARS-CoV-2 nucleic acid/antigen negativity; myocardial enzymes: myoglobin (Mb), creatine kinase (CK), creatine kinase-MB (CK-MB), lactate dehydrogenase (LDH), and α-hydroxybutyrate dehydrogenase (a-HBDH); liver function tests: alanine aminotransferase (ALT) and aspartate aminotransferase (AST); renal function tests: blood urea nitrogen (BUN) and serum creatinine (Scr); and thyroid function: TSH, FT3, FT4; thyroid autoantibody: TPOAb and TgAb.

#### Thyroid sonography

Upon admission, thyroid ultrasound examinations conducted by the Department of Medical Ultrasound at The Second Hospital of Hebei Medical University were analyzed.

#### Longitudinal monitoring

The longitudinal changes in TPOAb, TgAb, and thyroid function were monitored and analyzed in HT patients at1~ 3 and 3~6 months post-discharge.

### Statistical methods

All statistical analyses were performed using SPSS version 27.0 (IBM Corp). Continuous variables were reported as mean ± standard deviation (SD) if normally distributed, or median (interquartile range, IQR) if non-normally distributed. Categorical variables were expressed as frequencies (percentages). Group comparisons were conducted using independent t-tests or ANOVA for normally distributed continuous variables with homogeneous variances, and Mann-Whitney U or Kruskal-Wallis tests for non-normally distributed data. The chi-square test was used for categorical variables.

The outcome variable “HT detected during COVID-19 hospitalization” was coded as 1 = yes, 0 = no. Binary independent variables (e.g., sex, smoking history, comorbidities) were assigned 1 = yes, 0 = no, while continuous independent variables (e.g., age, viral clearance time, laboratory parameters) were included as raw data. Univariate and multivariate logistic regression analyses were performed to identify risk factors for COVID-19-associated newly diagnosed HT and mortality. Variables with P<0.05 in univariate analysis were entered into the multivariate model, with results presented as odds ratios (ORs) and 95% confidence intervals (CIs). The Hosmer-Lemeshow test (χ² = 6.23, P = 0.62) confirmed acceptable model fit, and all variance inflation factor (VIF) values < 2.5 indicated no multicollinearity. A two-tailed P-value <0.05 was considered statistically significant.

## Result

### Patient selection and flow

**Figure 2:**
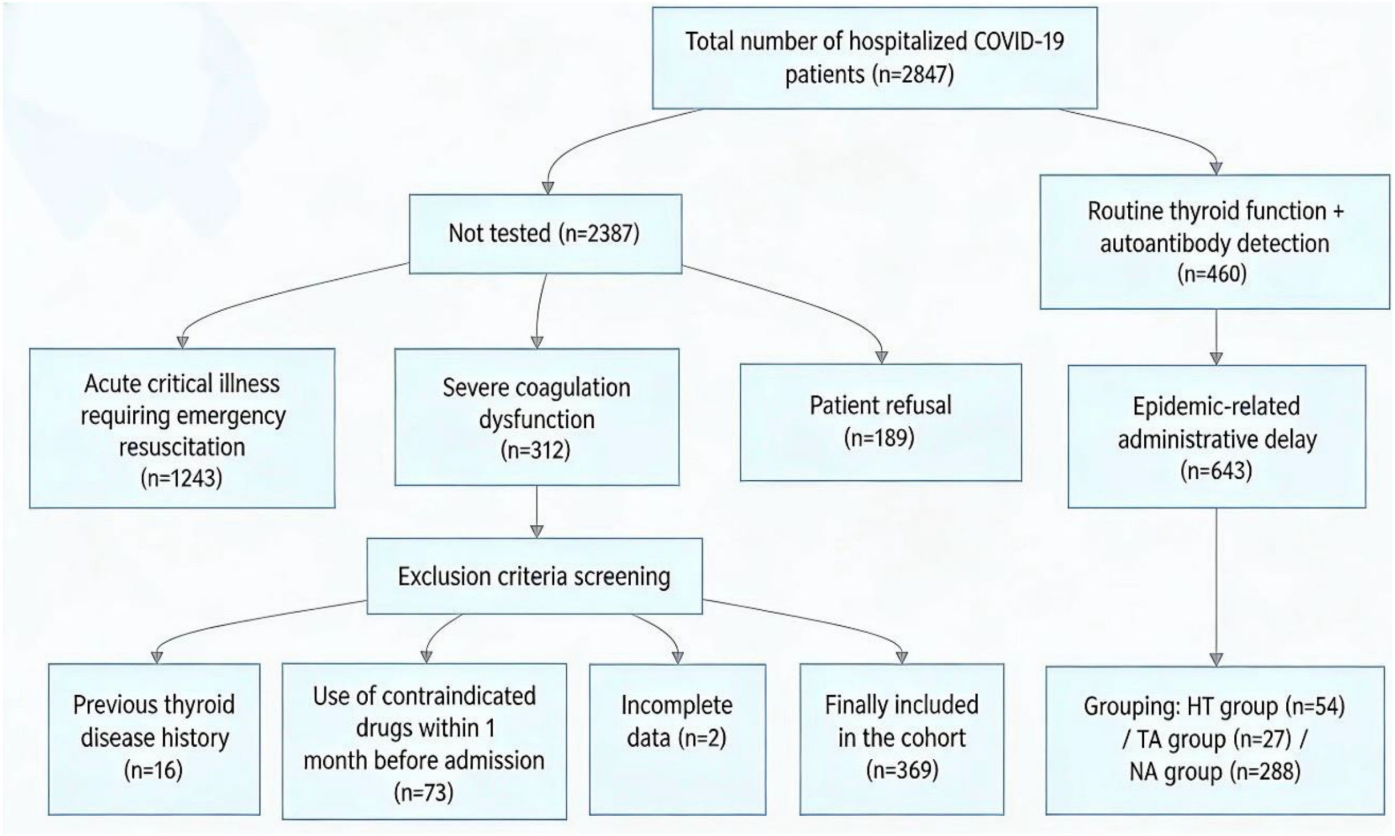
Patient selection and flow.

In this study, a total of 2847 COVID-19 patients were hospitalized during the study period. Among them, 460 patients underwent thyroid function and autoantibody testing (detection rate: 16.16%), with 91 patients excluded based on predefined criteria (16 with prior thyroid disease, 73 with contraindicated drug use, 2 with incomplete data), resulting in a final cohort of 369 patients. The reasons for non-testing in 2387 patients were: acute critical illness requiring emergency resuscitation(n=1243), severe coagulation disorders (n=312), patient refusal (n=189). (**Figure 2**).

### General characteristics of the HT, TA and NA group

The median age of the HT group (median: 72.5 years, IQR:63.5-79.0) was significantly higher than that of the NA group (median:69 IQR:58.3-76.0, P = 0.048). In contrast, no significant differences were detected between the HT and TA groups (median:73.0 years, IQR:67.0-81.0) or between the TA and NA groups. The prevalence of fatigue was significantly higher in the HT group compared to the TA and NA groups (53.7% vs. 22.2%, P < 0.001; 53.7% vs. 24.0%, P<0.007). (**Table 1**).

**Table 1:** Baseline demographics and clinical presentations in the HT, TA, NA groups.

Item	HT (n=54)	TA (n=27)	NA(n=288)	*P value*
Age (years)	72.5 (63.5,79.0) *	73.0 (67.0,81.0)	69.0 (58.3,76.0)	0.031
Male(n%)	26 (48.1)	14 (51.9)	175 (60.8)	0.176
BMI(Kg/m²)	24.3 ± 3.0	25.2 ± 3.0	24.9 ± 3.5	0.247
Smoking history(n%)	14 (25.9)	5 (18.5)	69 (24.2)	0.756
Comorbidities (n%)				
Hypertension	32 (59.3)	17 (63.0)	146 (50.9)	0.281
Coronary heart disease	15 (27.8)	10 (37.0)	69 (24.0)	0.301
Diabetes mellitus	10 (18.5)	7 (25.9)	87 (30.2)	0.208
COPD	6 (11.1)	3 (11.1)	27 (9.4)	0.908
Tumor	4 (7.4)	0 (0)	15 (5.2)	0.186
Symptom (n%)				
Fever	51 (94.4)	25 (92.6)	258 (89.6)	0.460
Cough/Sputum production	47 (87.0)	18 (66.7)	210 (72.9)	0.057
Dry throat/Pharyngodynia	9 (16.7)	6 (22.2)	29 (10.1)	0.117
Nasal congestion/Rhinorrhea	5 (9.3)	3 (11.1)	16 (5.6)	0.399
Nausea/Vomiting	5 (9.3)	4 (14.8)	24 (8.3)	0.573
Chest tightness/Dyspnea	25 (46.3)	13 (48.1)	111 (38.2)	0.363
Fatigue	29 (53.7)*** ^△^	6 (22.2)	69 (24.0)	<0.001
Myalgia	8 (14.8)	2 (7.4)	17 (5.6)	0.109
Headache	10 (18.5)	3 (11.1)	26 (9.0)	0.150
Diarrhea	1 (1.9)	0 (0)	6 (2.1)	0.581
Time to viral clearance (days)	6 (5,8)**^△^	4 (3,5)	4 (3,6)	0.004
Severe COVID-19 (n%)	21 (38.9)	10 (37.0)	78 (27.1)	0.147
Length of hospital stay (days)	10 (7,15)	8 (6,12)	9 (7.25,12)	0.233
ICU admission rate (n%)	8 (14.8)	4 (14.8)	26 (9.1)	0.328
Mortality (n%)	5 (9.3)	3 (11.1)	19 (6.6)	0.605

* indicates a statistically significant difference between the HT group and the NA group, P < 0.05; ** indicates a statistically significant difference between the HT group and the NA group, P < 0.01; *** indicates a statistically significant difference between the HT group and the NA group, P < 0.001;△ indicates a statistically significant difference between the HT group and the TA group, P < 0.01. (BMI, Body Mass Index; COPD, chronic obstructive pulmonary disease; ICU, Intensive Care Unit).

### Laboratory test results of the HT, TA, and NA groups

Among COVID-19 patients, the overall incidence of thyroid dysfunction was 42.0%, comprising: NTIS (18.4%), subclinical hypothyroidism (4.3%), hypothyroidism (2.4%), and subclinical hyperthyroidism (16.8%). No cases of overt hyperthyroidism were observed. The incidence of thyroid dysfunction was significantly higher in the HT group (57.4%) and TA group (48.1%) compared to the NA group (38.4%) (P = 0.029). The incidence of subclinical hyperthyroidism was significantly higher in the HT group compared to the NA group (29.6% *vs* 13.9%, P = 0.02) (**Table 2**).

**Table 2:** Laboratory test results of the HT group, TA group, and NA group.

Item	Reference range	HT(n=54)	TA(n=27)	NA(n=288)	*P value*
WBC(×10^9^/L)	3.5~9.5	6.3(4.8,9.7)	7.4(4.9,11.0)	6.6(5.1,8.7)	0.552
Rate of WBC Elevation (n%)		16(29.6)	8(29.6)	60(20.8)	0.249
NEUT(×10^9^/L)	2~7	4.3(3.2,8.2)	6.1(3.3,10.1)	4.9(3.3,6.8)	0.322
Rate of NEUT Elevation (n%)		21(38.9) *	10(35.9)	68(23.6)	0.031
LYMPH(×10^9^/L)	1.1~3.2	1.1(0.5,1.5)	0.8(0.5,1.3)	1.1(0.7,1.5)	0.243
Rate of LYMPH Reduction (n%)		31(57.4)	18(66.7)	154(53.5)	0.390
PLT(×10^9^/L)	125~350	206.5(122.0,257.5)	175.0(134.0,255.0)	207.5(154.0,266.3)	0.247
Rate of PLT Reduction (n%)		13(24.1)	4(14.8)	34(11.8)	0.056
NLR	0.9~4.0	4.5(2.3,9.9)	7.9(2.8,20.3)	4.7(2.8,8.8)	0.185
PLR	100~300	180.7(51.6,315.1)	194.2(131.5,374.0)	201.3(137.8,325.8)	0.600
IL-6 pg/mL	0~7	12.3(5.8,28.1)	6.1(3.6,16.7)	6.6(4.2,23.6)	0.119
Rate of IL-6 Elevation (n%)		18(58.1)	4(40.0)	58(41.7)	0.245
IL-17 pg/mL	0~19	5.0(4.3,5.6)	4.5 ± 1.4	4.5(4.0,5.4)	0.066
Rate of IL-17 Elevation (n%)		14(45.2)	3(30.0)	42(30.2)	0.286
TNF-a pg/mL	0~8	4.0(4.0,4.1)	3.8 ± 0.6	4.0(3.3,4.1)	0.056
Rate of TNF-a Elevation (n%)		12(38.7)	4(40.0)	35(25.2)	0.238
HsCRP ng/L	0~6	29.6(10.0,80.3)	18.5(3.2,193.8)	23.1(6.1,76.5)	0.581
Rate of HsCRP Elevation (n%)		45(83.3)	17(63.0)	217(75.6)	0.129
D-D μmol/L	0~0.2	0.3(0.2,0.5)	0.2(0.2,0.9)	0.2(0.1,0.4)	0.278
Rate of D-D Elevation (n%)		30(55.6)	13(48.1)	138(47.9)	0.585
Mb ng/mL	20~60	58.5(44.8,92.0)	88.0(38.3,169.0)	63.0(46.0,91.0)	0.359
CK U/L	38~140	49.0(34.0,77.5)	74.0(36.0,153.0)	56.0(37.3,90.8)	0.141
CK-MB U/L	0~18	15.0(10.8,23.3)	19.0(12.0,28.0)	15.0(11.0,20.0)	0.078
LDH U/L	120~250	241.5(190.25,299.75)	261(171,344)	213.5(170,268.75)	0.063
α-HBDH U/L	72~182	196.0(141.8,228.5)	204.4(128.0,293.0)	158.5(127.3,218.0)	0.058
ALT U/L	7~40	22.5(15.2,32.0)	24.0(14.0,49.3)	19.7(12.5,33.8)	0.428
AST U/L	13~35	22.9(19.0,31.0)	23.0(19.2,41.2)	22.0(16.0,31.6)	0.187
BUN mmol/L	3.1~8.8	5.2(4.5,7.4)	5.2(4.3,6.6)	5.2(4.0,6.8)	0.659
Scr μmol/L	41~81	62.0(49.8,76.3)	70.0(54.0,91.0)	70.0(55.0,85.0)	0.051
Myocardial Injury (n%)		5(9.3)	3(11.1)	20(6.9)	0.649
Liver Injury (n%)		6(11.1)	8(29.6)	54(18.8)	0.123
Kidney Injury (n%)		4(7.4)	6(22.2)	27(9.4)	0.082
TSH mIU/L	0.56~4.8	1.2(0.4,2.8)	1.2(0.4,2.1)	1.2(0.7,2.1)	0.734
FT3 pmol/L	3.5~6.5	3.6(3.2,4.2)	3.7(2.2,4.1)	3.8(3.4,4.4)	0.058
FT4 pmol/L	11.5~22.7	13.9(12.2,15.5)	14.0(12.2,16.5)	14.3(12.5,16.0)	0.556
Euthyroidism(n%)		23(42.6) *	14(51.9)	177(61.6)	0.029
Thyroid dysfunction(n%)		31(57.4)	13(48.1)	111(38.4)	0.029
NTIS(n%)		7(13.0)	4(14.8)	57(19.8)	0.413
Subclinical Hypothyroidism(n%)		4(7.4)	2(7.4)	10(3.5)	0.352
Hypothyroidism (n%)		4(7.4) *	0(0.0)	5(1.7)	0.032
Subclinical Hyperthyroidism(n%)		16(29.6) *	6(22.2)	40(13.9)	0.020

* indicates a statistically significant difference between the HT group and NA group (P<0.05); (WBC, White blood cell count; NEUT, Neutrophil Count; LYMPH, Lmphocyte Count; PLT, Platelet Count; NLR, Neutrophil-to-Lymphocyte Ratio; PLR, Platelet-to-Lymphocyte; hsCRP, High-sensitivity C-reactive protein; D-D, D-dimer; IL-6, Interleukin-6; IL-17, Interleukin-17; TNF-α, Tumor Necrosis Factor-alpha; Mb, Myoglobin; CK, Creatine kinase; CK-MB, Creatine kinase-MB; LDH, Lactate dehydrogenase; a-HBDH, α-Hydroxybutyrate Dehydrogenase; ALT, Alanine Aminotransferase; AST, Aspartate minotransferase; BUN, Blood Urea Nitrogen; Scr, Serum creatinine; TSH, Thyroid Stimulating Hormone; FT3, Free Triiodothyronine; FT4, Free Thyroxine; NTIS, Nonthyroidal Illness Syndrome).

### Comparison of inflammatory markers, thyroid function, and autoantibody profiles between mild and severe COVID-19 cases

The levels and percentage of WBC[8.3(5.9, 10.7)vs 6.3(4.9, 8.5)×10^9^/L, P<0.001;42.2% vs 14.6%, P<0.001], NEUT[6.1(4.2, 8.9)vs 4.4(3.1, 6.6)×10^9^/L, P<0.001;42.2% vs 20.4%, P<0.001], NLR[8.2(4.6, 17.5)vs 3.8(2.4, 7.3), P<0.001], PLR[258.6(163.8, 409.8)vs 184.4(129.5, 290.0), P<0.001], hsCRP[66.2(18.4, 151.2)vs 17.0(4.8, 54.2)ng/L, P<0.001; 86.2% vs71.4%, P = 0.002], D-D[0.4(0.2, 0.7)vs 0.2(0.1, 0.3)μmol/L, P<0.001;76.1% vs 37.7%, P<0.001], IL-6 [13.4(6.4, 30.8)vs 5.6(3.9, 16.2)pg/mL, P<0.001;60.3% vs 34.8%, P<0.001], IL-17 [5.3(4.3, 6.6)vs 4.3(3.8, 5.0)pg/mL, P<0.001;55.9% vs 18.8%, P<0.001] and TNF-α [4.1(4.0, 4.8)vs 3.8(3.2, 4.0)pg/mL, P<0.001;54.4% vs 12.5%, P<0.001]were significantly higher in the severe group compared to the mild group; The levels and percentage of PLT[192.0(130.5, 249.5)vs 210.5(157.0, 268.5)×10^9^/L, P<0.001;20.2% vs 11.2%, P<0.001] and LYMPH[0.8(0.4, 1.2)vs 1.1(0.7, 1.6)×10^9^/L, P<0.001;69.7% vs 48.8%, P<0.001] were significantly lower (P < 0.05). **Table 3**.

**Table 3:** Comparison of inflammatory biomarkers, thyroid function, and autoantibody levels between mild and severe COVID-19 patients.

Item	Mild group (n=260)	Severe group (n=109)	t/X2/Z	*P value*
WBC(×10^9^/L)	6.3(4.9,8.5)	8.3(5.9,10.7)	-4.650	<0.001
Rate of WBC Elevation (n%)	38(14.6)	46(42.2)	33.243	<0.001
NEUT(×10^9^/L)	4.4(3.1,6.6)	6.1(4.2,8.9)	-4.984	<0.001
Rate of NEUT Elevation (n%)	53(20.4)	46(42.2)	18.622	<0.001
LYMPH(×10^9^/L)	1.1(0.7,1.6)	0.8(0.4,1.2)	-5.310	<0.001
Rate of LYMPH Reduction (n%)	127(48.8)	76(69.7)	13.528	<0.001
PLT(×10^9^/L)	210.5(157.0,268.5)	192.0(130.5,249.5)	-2.026	0.043
Rate of PLT Reduction (n%)	29(11.2)	22(20.2)	5.257	0.022
NLR	3.8(2.4,7.3)	8.2(4.6,17.5)	-6.454	<0.001
PLR	184.4(129.5,290.0)	258.6(163.8,409.8)	-3.755	<0.001
IL-6 pg/mL	5.6(3.9,16.2)	13.4(6.4,30.8)	-4.830	<0.001
Rate of IL-6 Elevation (n%)	39(34.8)	41(60.3)	11.119	<0.001
IL-17 pg/mL	4.3(3.9,5.0)	5.3(4.3,6.6)	-5.120	<0.001
Rate of IL-17 Elevation (n%)	21(18.8)	28(55.9)	26.477	<0.001
TNF-α pg/mL	3.8(3.2,4.2)	4.1(4.0,4.8)	-6.575	<0.001
Rate of TNF-αElevation (n%)	14(12.5)	37(54.4)	36.603	<0.001
hsCRP ng/L	17.0(4.8,54.2)	66.2(18.4,151.2)	-6.463	<0.001
Rate of hsCRP Elevation (n%)	185(71.4)	94(86.2)	9.177	0.002
D-D μmol/L	0.2(0.1,0.3)	0.4(0.2,0.7)	-7.267	-7.267
Rate of D-D Elevation (n%)	98(37.7)	83(76.1)	45.445	<0.001
TSH mIU/L	1.4(0.8,2.7)	0.8(0.3,1.6)	-4.993	<0.001
FT_3_ pmol/L	4.0(3.5,4.5)	3.5(2.7,3.8)	-6.780	<0.001
FT_4_ pmol/L	14.0(12.4,15.9)	14.8(12.3,16.3)	-1.673	0.094
TPOAb IU/ml	28.0(28.0,41.0)	30.0(28.0,80.5)	-3.203	0.001
TgAb IU/ml	0.9(0.5,15.0)	1.0(0.6,15.0)	-1.575	0.115
TPOAb positivity rate (n%)	38(14.6)	29(26.6)	7.430	0.006
TgAb positivity rate (n%)	45(17.3)	23(21.1)	0.735	0.391
HT incidence (n %)	33(12.7)	21(19.3)	2.657	0.103
Euthyroidism(n%)	174(66.9)	39(35,8)	30.525	<0.001
Thyroid Dysfunction (n%)	86(33.1)	70(64.2)	30.525	<0.001
NTIS (n%)	35(13.5)	33(30.3)	14.444	<0.001
Subclinical Hypothyroidism(n%)	16(6.2)	0(0.0)	7.012	0.018
Hypothyroidism (n%)	5(1.9)	4(3.7)	0.147	0.534
Subclinical Hyperthyroidism(n%)	29(11.2)	33(30.3)	20.088	<0.001

The prevalence of thyroid dysfunction in COVID-19 patients is 42.0%, with the most prevalent subtypes being NTIS (18.4%), followed by subclinical hyperthyroidism (16.8%), subclinical hypothyroidism (4.3%), and overt hypothyroidism (2.4%). The prevalence of thyroid dysfunction was significantly elevated in the severe group compared to the mild group (64.2% vs 33.1%, P<0.001). Serum TSH and FT3 levels were significantly reduced in the severe group relative to the mild group [0.8 (0.3, 1.6)mIU/L vs 1.4 (0.8, 2.7)mIU/L, P<0.001; 3.5 (2.7, 3.8)pmol/L vs 4.0 (3.5, 4.5)pmol/L, P<0.001]. The levels and positivity rates of TPOAb were markedly elevated in severe cases compared with mild cases [30 (28, 80.5)IU/ml vs 28 (28, 41)IU/ml, P = 0.001; 26.6% vs 14.6%, P = 0.006]. (**Table 3**).

### Univariate and multivariate analysis of risk factors for newly diagnosed Hashimoto’s thyroiditis following COVID-19 infection

Univariate analysis demonstrated that the time to nucleic acid/antigen clearance (P<0.001) was significantly associated with HT. Multivariate logistic regression analysis revealed that associated with the detection of HT during COVID-19 hospitalization (OR = 1.136, P = 0.035). However, as this is an observational study, causality cannot be established (**Table 4**).

**Table 4:** Univariate and multivariate analysis of risk factors for newly diagnosed HT in COVID-19 patients.

	Univariate analysis		Multivariate analysis	
Item	OR (95% CI)	*P*	OR (95% CI)	*P*
Age (years)	1.019 (0.996~1.042)	0.107		
Male(n%)	1.615 (0.905~2.884)	0.105		
Time to viral clearance (days)	1.136 (1.009~1.278)	0.035	1.136 (1.009~1.278)	0.035
COVID-19 severity (n%)	1.642 (0.901~2.991)	0.105		
WBC(×10^9^/L)	1.058 (0.989~1.131)	0.099		
NEUT(×10^9^/L)	1.036 (0.963~1.115)	0.346		
LYMPH (×10^9^/L)	1.188 (0.896~1.573)	0.231		
NLR	1.006 (0.975~1.038)	0.704		
PLR	1.000 (0.999~1.001)	0.992		
hsCRP ng/L	1.000 (0.996~1.003)	0.821		
D-D μmol/L	0.895 (0.637~1.257)	0.521		
IL-6 pg/mL	1.006 (0.993~1.020)	0.382		
IL-17 pg/mL	1.000 (0.962~1.039)	0.998		
TNF-a pg/mL	1.134 (0.794~1.622)	0.439		

Variable coding: The outcome variable “HT detected during hospitalization for COVID-19” was coded as 1 = yes, 0 = no. Binary independent variables (e.g., sex, smoking history, comorbidities) were assigned as 1 = yes, 0 = no. Continuous independent variables (e.g., age, viral clearance time, laboratory parameters) were included as raw data. Model goodness-of-fit: Hosmer-Lemeshow χ² = 6.23, P = 0.62; all variance inflation factor (VIF) values were < 2.5, indicating no multicollinearity.

### Univariate and multivariate logistic regression analyses for risk factors of mortality

Univariate analysis demonstrated that advanced age (P<0.001), delayed viral clearance (P = 0.021), coronary heart disease (P = 0.007), and lower FT3 levels (P<0.001) were significantly associated with increased mortality risk, Multivariate logistic regression analysis revealed that while lower FT3 levels were independently associated with increased mortality risk (OR = 4.233, 95% CI 2.320–7.725, P<0.001), the inverse association observed for advanced age (OR = 0.944, 95% CI 0.896–0.994, P = 0.030) likely reflects more aggressive clinical management in severely ill elderly patients rather than a true protective effect (**Table 5**).

**Table 5:** Univariate and multivariate logistic regression analyses of factors associated with COVID-19 mortality.

	Univariate analysis		Multivariate analysis	
Items	OR (95% CI)	*P*	OR (95% CI)	*P*
Age (years)	0.904 (0.862~0.948)	<0.001	0.944 (0.896~0.994)	0.030
Male(n%)	1.769 (0.754~4.153)	0.190		
Time to viral clearance (days)	0.841 (0.727~0.974)	0.021	0.917 (0.764~1.099)	0.347
Hypertension(n%)	0.536 (0.234~1.227)	0.140		
Coronary heart disease (n%)	0.334 (0.151~0.740)	0.007	0.599 (0.240~1.494)	0.271
Diabetes mellitus (n%)	0.460 (0.208~1.019)	0.056		
COPD (n%)	0.597 (0.194~1.835)	0.368		
Tumor (n%)	1.453 (0.187~11.323)	0.721		
TSH (mIU/L)	0.976 (0.950~1.004)	0.089		
FT3 (pmol/L)	5.805 (3.267~10.314)	<0.001	4.233 (2.320~7.725)	<0.001
FT4 (pmol/L)	1.147 (0.998~1.319)	0.053		
TgAb (U/mL)	0.775 (0.300~2.000)	0.598		
TPOAb (U/mL)	0.608 (0.246~1.502)	0.281		
HT comorbidity status (n%)	0.736 (0.266~2.035)	0.544		

Note: Variable coding: The outcome variable “in-hospital mortality” was coded as 1 = death, 0 = survival. Coding for other variables was identical to that in **Table 4**. Model goodness-of-fit: Hosmer-Lemeshow χ² = 8.15, P = 0.42; all variance inflation factor (VIF) values were < 2.5, indicating no multicollinearity. The univariate inverse association with coronary heart disease may be affected by confounding factors and does not support a protective effect against mortality.

### Dynamic changes in thyroid profiles among hospitalized HT patients from admission to post-discharge follow-up

Among the 54 patients in the HT group, thyroid function and antibody levels were reassessed in 14 patients at1~ 3 months post-discharge. Of these, 6 patients (42.86%) exhibited thyroid dysfunction upon admission, while 3 patients (21.43%) had persistent dysfunction at discharge. Both TPOAb and TgAb were positive in all patients at admission. At discharge, 10 patients (71.4%) remained antibody-positive, whereas 4 patients (28.6%) seroconverted to negative (**Table 6**).

**Table 6:** Thyroid function and antibody levels at admission, 3 months, and 6 months post-discharge.

No.	At admission					1~3-Month follow-up					3~6-Month follow-up				
	TPOAb	TgAb	TSH	FT_3_	FT_4_	TPOAb	TgAb	TSH	FT_3_	FT_4_	TPOAb	TgAb	TSH	FT_3_	FT_4_
1	>1300	500.0	0.1	3.5	14.7	>1300	>500	0.2	17.0	17.8	–	–	–	–	–
2	216	342.7	3.7	3.9	13.2	1.5	15.0	3.7	4.7	9.6	–	–	–	–	–
3	165	134.4	10.0	4.4	15.2	28.0	0.5	8.1	4.8	14.9	57	0.8	6.9	4.7	17.6
4	>1300	69.0	1.9	3.1	11.4	>1300	355.0	2.3	5.8	7.6	>1300	157.3	0.7	5.0	11.8
5	215	68.5	2.0	4.2	12.1	0.3	5.0	2.2	4.1	12.5	–	–	–	–	–
6	195	600.0	0.4	3.8	16.0	24.2	17.2	2.0	5.4	19.4	–	–	–	–	–
7	252	472.0	1.3	3.3	8.9	252.0	472	20.5	3.3	8.9	–	–	–	–	–
8	215	>1000	0.4	3.5	11.5	75.0	106.7	0.7	3.7	11.6	0.3	<0.9	1.5	4.3	14.1
9	451	563.0	2.3	5.5	11.6	>1300	500.0	9.9	5.5	11.1	–	–	–	–	–
10	>1300	122.0	<0.1	6.1	17.9	>1300	276.0	0.9	3.8	8.1	472.0	NA	0.3	4.3	12.4
11	265	184.8	2.3	4.5	14.2	253.0	500.0	2.3	5.0	13.4	–	–	–	–	–
12	113	240.0	1.1	5.5	16.2	113.0	253.3	1.1	5.5	16.2	–	–	–	–	–
13	119	172.0	0.4	2.7	11.7	178.0	201.3	4.0	4.1	10.1	2.2	21.8	2.9	4.6	12.6
14	>1300	80.8	2.3	4.5	13.7	113.0	253.3	1.1	5.5	16.2	–	–	–	–	–

Thyroid ultrasound was performed in only 14/54 (25.9%) HT patients. Longitudinal follow-up data for thyroid function and antibodies were available for a subset of patients (n=14) at 1–3 and 3–6 months post-discharge, limiting generalizability.

## Discussion

In the present study, we conducted a retrospective cohort analysis of 369 hospitalized COVID-19 patients to explore the clinical characteristics, risk factors, and outcomes of newly diagnosed HT during acute SARS-CoV-2 infection.

HT is the most prevalent AITD and a leading cause of hypothyroidism in iodine-sufficient regions (6),. HT demonstrates an annual incidence rate of 0.3-1.5 cases per 1,000 individuals, with a striking 4–10 fold higher prevalence in women compared to men (30). The condition exhibits an age-dependent pattern, with peak antibody positivity occurring between 45–55 years (30). Furthermore, distinct ethnic variations exist, with Caucasians showing higher susceptibility than Black populations (5). Prior to the COVID-19 pandemic, the prevalence of H among individuals undergoing routine health examinations in China was approximately 10-12% (31). While multiple case reports have documented newly diagnosed HT following COVID-19 infection (14–23), comprehensive epidemiological studies investigating COVID-19-associated HT remain scarce. Current literature primarily focuses on post-COVID-19 alterations in thyroid function and autoantibody levels (12). In a notable study by Jia Di et al. (32), the incidence of HT among COVID-19 patients without pre-existing thyroid disease was 15.61% (325/2082), with stratification by disease severity revealing incidence rates of 12.34% (257/1625) in mild cases, 2.07% (43/208) in moderate cases, and 1.20% (25/179) in severe cases. In the present study, the overall incidence of HT among COVID-19 patients was 14.6%, significantly higher than the pre-pandemic prevalence (10-12%) observed in healthy Chinese populations (31). Stratified analysis revealed HT incidence rates of 8.9% (33/260) and 5.6% (21/109) in mild/moderate, and severe/critical COVID-19 cases, respectively. These findings align with the epidemiological patterns reported by Jia Di et al. (32), supporting the hypothesis that SARS-CoV-2 infection may be associated with newly diagnosed HT.

In this study, the male-to-female ratio among 54 HT patients was 1:1.04. The median age was 72.5(63.5, 79) years, suggesting that the male-to-female ratio in COVID-19-related HT was comparable, with a predominance of older adults. Patients with HT in the acute phase of COVID-19 presented with the following symptoms: fever (94.4%), cough/sputum production (87.0%), dry/sore throat (16.7%), nasal congestion/rhinorrhea (9.3%), nausea/vomiting (9.3%), chest tightness/dyspnea (46.3%), fatigue (53.7%), and myalgia (14.8%). These symptoms were consistent with the general clinical manifestations of COVID-19 in China (33) and lacked specificity. Compared to the TA (22.2%) and NA (24.0%) groups, the HT group (53.7%) exhibited significantly more pronounced fatigue (P < 0.001). This symptom may be associated with HT, it lacks specificity for HT diagnosis as fatigue is also a common manifestation of COVID-19 infection itself (34). To date, 11 cases of COVID-19-related HT have been documented in the literature (14–23). Among these, 3 cases (27.2%) were asymptomatic, while the remaining 8 cases (72.7%) manifested varioussymptoms including fatigue, alopecia, constipation, xerosis, mood disturbances, cervical discomfort, myasthenia, and weight gain. Our findings demonstrate that HT symptoms during acute COVID-19 infection show significant overlap with typical COVID-19 manifestations, creating diagnostic challenges in the early disease phase. Notably, fatigue emerges as a relatively specific indicator of concomitant HT, which should alert clinicians to consider prompt thyroid autoantibody testing for early HT detection.

The overall incidence of thyroid dysfunction in patients with COVID-19 in this study was 42.0%, with NTIS being the predominant manifestation (18.4%). Consistent with our findings, Ahn J et al. (35) reported thyroid dysfunction in 63.9% (76/119) of COVID-19 patients, with NTIS being most prevalent (18.5%), followed by subclinical hyperthyroidism (14.3%). This concordance between studies strengthens the evidence for SARS-CoV-2-mediated thyroid dysfunction, likely through multiple mechanisms including systemic inflammation, direct viral effects on thyroid tissue, and hypothalamic-pituitary-thyroid axis disruption. Early-stage HT exhibits significant heterogeneity in thyroid function profiles, potentially presenting as hyperthyroidism, hypothyroidism, or euthyroidism (36).

In this study, the incidence of thyroid dysfunction in the severe COVID-19 group (64.2%) was significantly higher than that in the mild COVID-19 group (33.1%) (P < 0.001). In the severe group, the most common thyroid dysfunction was subclinical hyperthyroidism (30.3%) and NTIS (30.3%). In the mild group the most common thyroid dysfunction was NTIS (13.5%). In the severe group, TSH levels 0.8(0.3, 1.6) mIU/L and FT3 levels 3.5(2.7, 3.8) pmol/L were significantly lower than those in the mild group. These findings suggest that patients with severe COVID-19 exhibit significantly lower TSH and FT3 levels compared to those with mild symptoms.

The HT group exhibited a significantly higher incidence of thyroid dysfunction (57.4%) compared to the TA (51.9%) and NA (38.4%) groups (P = 0.021). The predominant thyroid dysfunction subtypes included subclinical hyperthyroidism (29.6%), NTIS (13.0%), hypothyroidism (7.4%), and subclinical hypothyroidism (7.4%). Notably, subclinical hyperthyroidism occurred more frequently in the HT group than in the TA and NA groups. HT is the most prevalent cause of hypothyroidism (6). However, thyroid function in HT is heterogeneous and does not invariably present as hypothyroidism. In early-stage HT, patients may exhibit hyperthyroidism or euthyroidism (34). Progressive destruction of thyroid follicular cells ultimately leads to hypothyroidism. However, this process exhibits marked interindividual variability due to the potential intermittency of thyroid cell destruction (37). Studies have demonstrated that transient hyperthyroidism is a hallmark feature in COVID-19 patients with AITD (38), consistent with our findings. These findings suggest that HT patients in the acute phase of COVID-19 are more likely to develop subclinical hyperthyroidism than hypothyroidism.

Although patients with preexisting HT demonstrated a markedly higher rate of thyroid dysfunction during acute COVID-19 than those without HT, it is critically difficult to differentiate alterations specifically caused by underlying AITD from transient changes related to NTIS in the setting of acute severe systemic infection. The observed pattern of thyroid dysfunction, particularly subclinical hyperthyroidism, in hospitalized HT patients may be multifactorial. Potential contributors include the acute phase response of COVID-19 (NTIS), direct or inflammatory effects of SARS-CoV-2 on the thyroid gland (39), and persistent autoimmune activity associated with HT itself (13). Accordingly, overinterpretation of these abnormalities as exclusive manifestations of HT should be avoided, as NTIS related to acute infection can overlap with or mask typical HT-related thyroid function changes (40).

TPOAb and TgAb serve as established markers of thyroid autoimmunity. Elevated TPOAb or TgAb levels represent significant indicators of thyroid autoimmunity (TA) and potential progression to HT (28). TPOAb titers demonstrate a direct correlation with progression risk to hypothyroidism, while TgAb exhibits superior diagnostic specificity for HT (41). During pre-hypothyroid stages, the presence of both TPOAb and TgAb serves as the sole diagnostic criterion for HT (6).

In this study, both the positivity rate and serum levels of TPOAb were significantly elevated in severe/critical COVID-19 patients compared to mild cases, whereas TgAb levels and positivity rates did not differ significantly across disease severities. These findings suggest that TPOAb elevation during acute COVID-19 may correlate with disease severity. Both TPOAb and TgAb levels were significantly elevated in the HT group compared to the TA and NA groups (P<0.001), indicating robust thyroid autoimmunity in COVID-19 patients with HT. SARS-CoV-2 proteins exhibit structural and antigenic homology with TPO, potentially inducing immune cross-reactivity that promotes thyroid autoimmunity against TPO (10).

In this study, patients with severe COVID-19 exhibited a marked systemic inflammatory response, with alterations in inflammatory markers and cytokine levels correlating with disease severity. These results align with previous reports (11, 13). The HT group demonstrated a significantly higher rate of neutrophilia (38.9%) compared to both the TA (35.9%) and NA (23.6%) groups (P = 0.031). Activated neutrophils in COVID-19 express NETs-related genes, engaging with innate immunity and interacting with T, NK, and B cells (42). Through NETs, neutrophils contribute to immune system hyperactivation and autoimmune responses (13). The absence of significant elevation in other inflammatory markers among the hypertension group suggests that SARS-CoV-2 may contribute to thyroid pathology through specific immune pathways (e.g., NETs-mediated autoimmunity) rather than systemic inflammatory activation.

The most prevalent underlying comorbidities among 54 HT patients were hypertension (59.3%), coronary heart disease (27.8%), diabetes mellitus (18.5%), and COPD (11.1%), tumor (7.4%). Several studies have suggested an association between HT and type 2 diabetes (43). Furthermore, HT has been linked to autoimmune diseases, including type 1 diabetes, rheumatoid arthritis, and systemic lupus erythematosus (SLE), collectively classified as autoimmune polyendocrine syndromes (44). Elevated levels of thyroid autoimmune antibodies (TPOAb and TgAb) have been observed in COVID-19 patients with comorbid type 2 diabetes. It has been hypothesized that hyperglycemia and insulin resistance could exacerbate virus-induced immune dysregulation, potentially enhancing thyroid autoantibody production (13). In contrast, a separate study reported reduced thyroid autoimmune antibody levels in COVID-19 patients with diabetes compared to those without diabetes (45).

The prevalence of severe and critical COVID-19 in the HT group (38.9%) did not differ significantly from the TA (37.0%) and NA (27.1%) groups (P = 0.147). Notably, all three groups demonstrated higher rates of disease severity compared to the national averages reported by China CDC (severe cases: 14%; critical cases: 5%) (46). Additionally, the mortality rate in the HT group (9.3%) showed no significant difference compared to the TA (11.1%) and NA (6.6%) groups, but exceeded the 2.3% national COVID-19 mortality rate reported by China CDC (46). This elevated mortality may reflect our study’s inclusion criteria, which were limited to hospitalized patients with disease progression. The median hospitalization duration in the HT group was 10 (7, 15) days, showing no statistically significant difference compared to the TA [8 (6, 12) days] and NA [9 (7.3, 12) days] groups (P = 0.233). This was marginally shorter than the national average of 14 days for COVID-19 hospitalizations in China (47), potentially reflecting regional variations in admission and discharge protocols. Importantly, our analysis revealed no significant differences between the HT group and other groups regarding disease severity, mortality rates, or length of hospital stay.

For the risk factors of newly diagnosed HT, Multivariate logistic regression analysis revealed that prolonged SARS-CoV-2 nucleic acid/antigen clearance time (OR = 1.136, 95%CI 1.009~1.278, P = 0.035) is an independent factor associated with the identification of newly diagnosed HT during COVID-19 hospitalization. This association supports the hypothesis that sustained viral replication may contribute to the development or unmasking of thyroid autoimmunity, possibly through mechanisms such as immune cross-reactivity or persistent inflammatory stimulation (48). However, no significant associations were observed between HT and gender, age, inflammatory markers, or cytokine levels, indicating that the link between COVID-19 and thyroid autoimmunity may be specific to viral persistence rather than general systemic inflammation (13).

Among the COVID-19 cohort in this study, 27 fatalities were recorded, of which 20 exhibited thyroid dysfunction, including 12 cases of NTIS, 2 cases of hypothyroidism, and 6 cases of subclinical hyperthyroidism. Univariate analysis demonstrated that advanced age (P<0.001), delayed viral clearance (P = 0.021), coronary heart disease (P = 0.007), and lower FT3 levels (P<0.001) were significantly associated with mortality risk. However, multivariate logistic regression analysis revealed distinct associations: advanced age (OR = 0.944, 95%CI 0.896~0.994, P = 0.030) was associated with decreased mortality risk, which may be attributed to potential confounding factors such as more aggressive clinical management for elderly patients with severe COVID-19 in this cohort. In contrast, lower FT3 levels (OR = 4.233, 95%CI 2.320~7.725, P<0.001) remained an independent predictor of mortality, consistent with previous studiesindicating that reduced FT3 reflects severe systemic inflammation and impaired metabolic function, which are closely linked to adverse outcomes in COVID-19 (49). Notably, delayed viral clearance showed a significant association with mortality in univariate analysis (P = 0.021) but failed to retain statistical significance in the multivariate model (OR = 0.917, P = 0.347), suggesting that its impact on mortality may be mediated by other factors such as disease severity or inflammatory responses. The negative correlation between coronary heart disease and mortality in univariate analysis (OR = 0.334, P = 0.007) should be interpreted cautiously, as it may result from limited sample size or selection bias (more rigorous monitoring and intervention for COVID-19 patients with preexisting coronary heart disease), rather than a true protective effect.

Jafarzadeh A et al. (50) summarized findings from 83 reported cases and found that subacute thyroiditis (SAT) was the most common COVID-19 vaccination-associated thyroid disorder, accounting for 60.2% of cases, followed by Graves’ disease (GD) at 25.3%. Lui DTW et al. (51) observed changes in thyroid function and antibody levels at baseline and 8 weeks after the second dose of a COVID-19 vaccine in 215 patients. Only 3 patients (1.4%) developed thyroid dysfunction post-vaccination. Post-vaccination anti-TPO and anti-Tg titers showed a modest increase, but these changes were not statistically significant. In the present study, all patients had received at least two doses of a COVID-19 vaccine prior to hospital admission, while the impact of COVID-19 vaccines on thyroid autoimmunity remains controversial.

The pathogenesis of COVID-19-associated HT is likely multifactorial, involving direct viral effects, immune dysregulation, and molecular mimicry (32). Thyroid follicular epithelial cells express angiotensin-converting enzyme 2 (ACE2) and its cofactor transmembrane protease serine 2 (TMPRSS2), providing a structural basis for SARS-CoV-2 invasion (13). Viral infection may directly damage thyroid tissue, releasing thyroid antigens and triggering autoimmune responses. Additionally, SARS-CoV-2-induced cytokine storms (e.g., elevated IL-6, IL-17, and TNF-α) can exacerbate immune dysregulation, promoting the development of autoimmunity (39, 52).

Molecular mimicry between SARS-CoV-2 S protein and TPO is another key mechanism (10). TPOAb is the most specific marker of HT, and its levels correlate with the risk of progression to hypothyroidism (5). In our study, severe COVID-19 patients had significantly higher TPOAb levels and positivity rates than mild cases (P = 0.001 and P = 0.006, respectively), suggesting that TPOAb elevation may be associated with COVID-19 severity. Sustained SARS-CoV-2 replication, as reflected by prolonged viral clearance time, may stimulate continuous production of anti-S proteins, which cross-react with TPO and exacerbate thyroid autoimmunity (10).

It is important to note that the high prevalence of subclinical hyperthyroidism and low FT3 levels in HT patients may also reflect NTIS, a transient thyroid function disturbance associated with severe systemic illness (49, 53) Distinguishing HT-related thyroid changes from NTIS is a clinical challenge, as their laboratory features may overlap. Future prospective studies incorporating pre-infection thyroid function data are needed to disentangle the relative contributions of these two processes.

Longitudinal follow-up of 14 HT patients (25.9% of the HT cohort) revealed that 42.86% (6/14) had thyroid dysfunction at admission, while 21.43% (3/14) had persistent dysfunction at 1~3 months post-discharge. Notably, 28.6% (4/14) of patients seroconverted to TPOAb/TgAb-negative, suggesting that thyroid autoimmunity may be transient in some cases. However, 71.4% (10/14) remained antibody-positive, and 14.3% (2/14) progressed to overt hypothyroidism, consistent with previous studies showing that thyroid autoantibody abnormalities often persist despite the resolution of thyroid function disturbances (54).

Thyroid ultrasound, performed in 14 HT patients, revealed 100% pathological findings, including diffuse enlargement, nodular formations, and heterogeneous echogenicity—features characteristic of HT (5). These findings support the diagnosis of HT but also highlight the limitation of incomplete imaging data in our cohort, which may have affected the accuracy of HT diagnosis in patients without ultrasound confirmation.

In this study, 9 COVID-19 patients were diagnosed with hypothyroidism upon admission (4 in the HT group, and 5 in the NA group). All patients received levothyroxine replacement therapy. One patient died during hospitalization due to respiratory failure, while the remaining patients showed clinical improvement. These findings suggest that acute SARS-CoV-2 infection may induce thyroid dysfunction through multiple mechanisms (55), even in patients without pre-existing thyroid disease. Hypothyroidism secondary to COVID-19 could adversely affect prognosis and, in severe cases, pose life-threatening risks. To our knowledge, only 11 cases of COVID-19 complicated by HT have been reported in the literature (14–23). Notably, all developed hypothyroidism requiring levothyroxine replacement therapy. One patient succumbed to respiratory and cardiac arrest secondary to myxedema, while the remaining patients showed clinical improvement. Our findings align with these previously reported cases. These observations underscore the importance of thyroid function monitoring during acute COVID-19 infection. Timely initiation of levothyroxine replacement therapy in patients who develop hypothyroidism may significantly improve clinical outcomes.

Collectively, these results underscore the need for close monitoring of thyroid function and autoantibody levels in COVID-19 patients with pre-existing HT, particularly during the acute phase and subsequent recovery. Given the exploratory nature of our findings due to limited imaging and follow-up data, future studies with larger, well-characterized cohorts.

## Limit

This study has several limitations that should be acknowledged. First, as a retrospective cohort study, we lacked pre-COVID-19 thyroid function and autoantibody data, which prevented us from definitively distinguishing between *de novo* HT triggered by SARS-CoV-2 and previously undiagnosed pre-existing HT. Thus, our findings describe “newly diagnosed HT” identified during COVID-19 hospitalization rather than confirmed new-onset disease. Second, thyroid autoantibody testing was not performed in all hospitalized COVID-19 patients (detection rate: 16.16%), which may have introduced selection bias. Third, the sample size for longitudinal follow-up was small (n=14), limiting the generalizability of our findings on dynamic changes in thyroid function and autoantibodies. Fourth, fine-needle aspiration cytology (FNAC) was not performed in any patients, which may have affected the accuracy of HT diagnosis in cases with atypical ultrasound findings. Finally, we did not collect data on SARS-CoV-2 viral load, which prevented us from assessing its association with HT.

## Conclusions

COVID-19 patients complicated by HT are typically older, with fatigue being a distinguishing clinical manifestation. They are more prone to thyroid dysfunction, with subclinical hyperthyroidism being the predominant early thyroid function abnormality. Prolonged viral clearance time was identified as a factor associated with newly diagnosed HT.These findings highlight the need for routine thyroid function screening in high-risk COVID-19 patients and long-term follow-up of those with thyroid autoantibody positivity. Future prospective studies with pre-infection thyroid data and larger follow-up cohorts are warranted to confirm these observations and clarify the long-term impact of COVID-19 on thyroid health.

## Data Availability

The raw data supporting the conclusions of this article will be made available by the authors, without undue reservation.
